# Inappropriate Subcutaneous Implantable Cardioverter Defibrillator Shocks Secondary to Cardiac Remodeling: A Unique Case of T Wave Oversensing

**DOI:** 10.7759/cureus.26129

**Published:** 2022-06-20

**Authors:** Maleeha Saleem, Karan Pahuja, Tehreem Fatima, Steven Hamilton, Christina Wjasow, Justin Fox

**Affiliations:** 1 Internal Medicine, Saint Francis Medical Center, Trenton, USA; 2 Internal Medicine, Jersey Shore University Medical Center/Saint Francis Medical Center Program, Trenton, USA; 3 Electrophysiologist, Saint Francis Medical Center/Hamilton Cardiology Associates, Trenton, USA; 4 Cardiology, Saint Francis Medical Center, Trenton, USA

**Keywords:** anti tachycardia pacing, overdrive pacing, non ischemic cardiomyopathy, subcutaneous implantable cardioverter defibrillators, ventricular fibrillation

## Abstract

Implantable cardioverter defibrillators (ICD) are used for the primary and secondary prevention of sudden cardiac death (SCD). Currently, two different modalities of ICDs are in use: transvenous (TV) and subcutaneous (S-ICD). The use of S-ICDs has been driven by several potential benefits of this technology: preservation of central venous vasculature, no risk of vascular or myocardial injury during implant, easier explanation, and lower risk of systemic infections. Inappropriate shocks are defined as shocks delivered for non-life-threatening arrhythmias or because of oversensing. Here, we present a case of a 58-year-old man who began experiencing inappropriate shocks three years after S-ICD placement. Careful analysis of the ICD showed T wave oversensing with no malfunction of the device. The shocks persisted even after reprogramming, leading to subsequent ICD removal and loop recorder implantation. The onset of shock episodes coincided with the improvement of left ventricular ejection fraction (LVEF). To the best of our knowledge, this is the first published report of cardiac remodeling leading to uncorrectable T wave oversensing that subsequently required S-ICD explant. This represents a potentially important limitation of S-ICD technology, especially as S-ICD use rises and medical therapy for cardiomyopathy continues to improve.

## Introduction

Ventricular fibrillation (VF) is a common cause of sudden cardiac death (SCD), and the only effective approach for terminating VF is electrical defibrillation. In 1980, the successful use of external defibrillators led to the development of implantable cardioverter defibrillators (ICDs). Initially, ICDs were studied and approved for use for secondary prevention in survivors of cardiac arrest [1]. Subsequently, clinical trials identified those subsets of cardiomyopathy patients that benefit from ICD therapy for the primary prevention of SCD [2]. There are two main types of defibrillators in use currently: transvenous ICD (traditional) and subcutaneous ICD (S-ICD). Compared with traditional ICDs, S-ICDs are increasingly being considered in patients who do not require pacemaker functionality from their device for cardiac resynchronization or bradycardia and also in those who do not have documented monomorphic ventricular tachycardia (VT) that would be amenable to antitachycardia pacing therapies. S-ICDs are preferred in patients who are on hemodialysis or have an increased risk of infections in which preservation of the vasculature is of particular importance [3]. Approximately 10-20% of S-ICD patients experience inappropriate ICD shocks [4]. Inappropriate shocks are defined as shocks delivered for non-life-threatening arrhythmias or because of oversensing [5]. It is of utmost importance to identify the etiology of inappropriate shocks as it is associated with an increased risk of all-cause mortality [6]. Cases of oversensing can be subdivided into physiological oversensing such as T wave oversensing or double counting of QRS complexes, and non-physiological oversensing such as electromagnetic interference, lead fracture noise [6], generator faults, hypermobility of ICD generator, and subcutaneous residual air [7]. This research study presents an unusual case of inappropriate S-ICD shocks three years after implantation due to T wave oversensing in the setting of left ventricular ejection fraction (LVEF) improvement due to cardiac remodeling with subsequent change in vector sensing.

The abstract for this case report has been accepted as a poster presentation on June 14, 2022, at South/Central Region Resident /Fellow Physician Research day at Hackensack Meridian Health, School of Medicine.

## Case presentation

**Figure 1:**
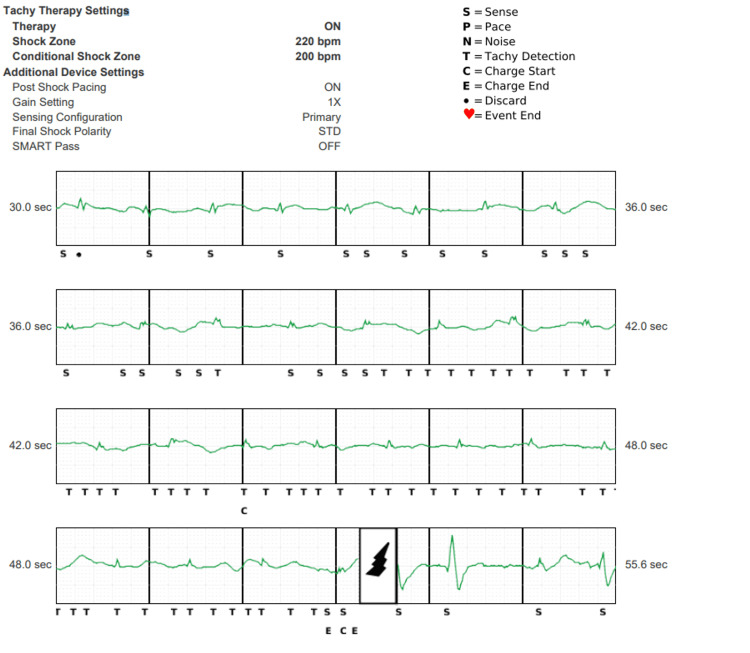
Interrogation of the S-ICD with settings shown in the image at the time of shock Vector setting was primary. Intracardiac electrograms from S-ICD showing initial double-counting of QRS and T waves that triggered device charging as denoted by C. At 52 sec, the device delivers a shock. Note the Smart Pass filter that was programmed on at implant was automatically disabled by the device and subsequently programmed off. S-ICD - subcutaneous implantable cardioverter defibrillator

A 58-year-old male with a medical history significant for aortic stenosis status post transaortic valvular replacement (TAVR) in 2016, non-ischemic cardiomyopathy with recovered LVEF, atrial flutter status post-ablation in 2019, hypertension, first degree atrioventricular (AV) block with right bundle branch block (RBBB) and left anterior fascicular block, hyperlipidemia, status post Boston Scientific Emblem MRI S-ICD model number A219 placed on 4/9/2018 came to the emergency room (ER) with a presenting complaint of ICD discharge. There were a total of three discharges that occurred while the patient was driving. He did not note chest pain, shortness of breath, palpitations, or dizziness. Lab results demonstrated significant hypokalemia (potassium of 3.1 [3.4-4.7 mmol/L]) and hypomagnesemia (magnesium of 1.6 [1.9-2.7mg/dL]). His EKG showed normal sinus rhythm with first-degree AV block, RBBB with left anterior fascicular block, and no change from the prior EKG. Interrogation of his S-ICD revealed three inappropriate shocks due to T wave oversensing, and no significant arrhythmias were noted, as shown in Figure 1.

A chest X-ray (CXR) demonstrated the implanted ICD with its lead unchanged in position from implantation. After reviewing all the possible etiologies, it was hypothesized that significant hypokalemia and hypomagnesemia may have changed the morphology of the electrocardiograms for arrhythmia sensing. The device was functionally normal, and in order to avoid double-counting, the sensing vector was switched to secondary with the Smart Pass filter turned back on. The patient was observed overnight on telemetry, and no significant arrhythmias were noted; the S-ICD was reprogrammed, and the patient was discharged home with appropriate electrolyte repletion therapy. 

**Figure 2:**
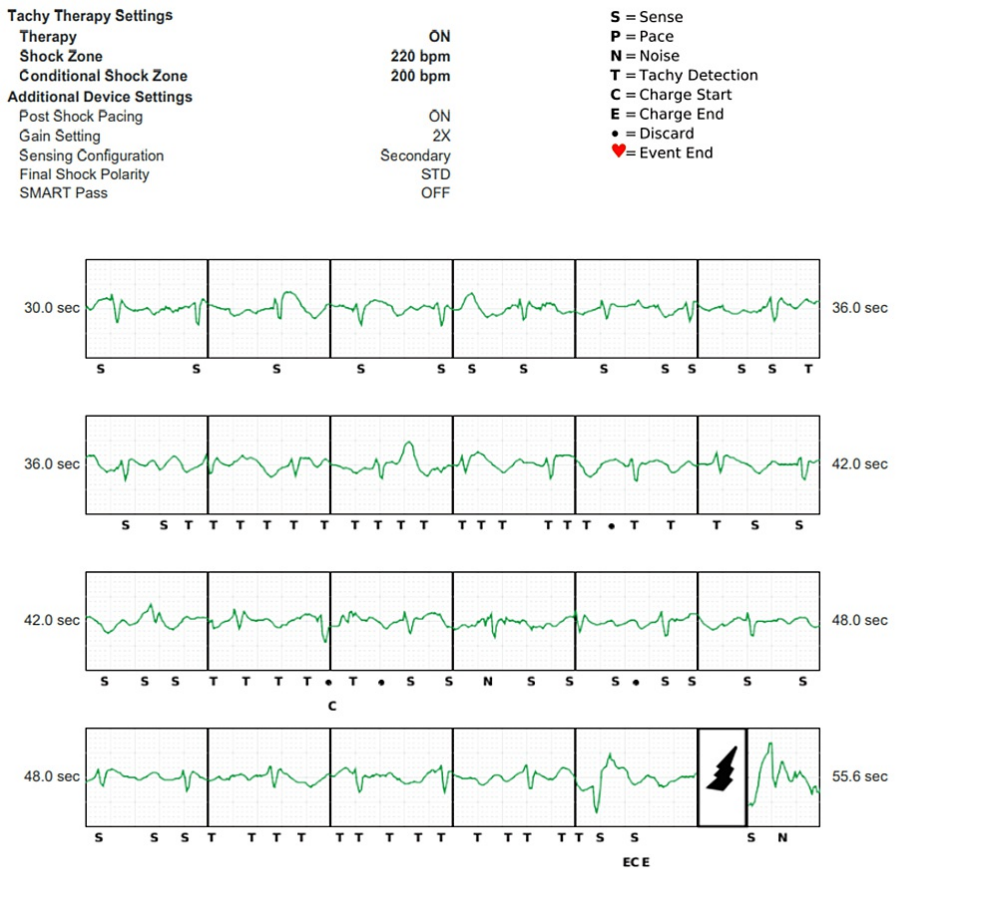
ICD settings and intracardiac electrograms Of note, despite the vector being changed to secondary, the Smart Pass filter was again automatically deactivated by the device, which then over-sensed T waves that ultimately led to the device falsely sensing it as tachycardia and inappropriately discharging. ICD - implantable cardioverter defibrillator

However, three months later, the patient again presented with two S-ICD discharges with stable electrolytes. The device was interrogated, and these ICD discharges were again found to be inappropriate due to oversensing of the T waves triggered in part by the device's automatic deactivation of the Smart Pass filter (Figure 2).

**Figure 3:**
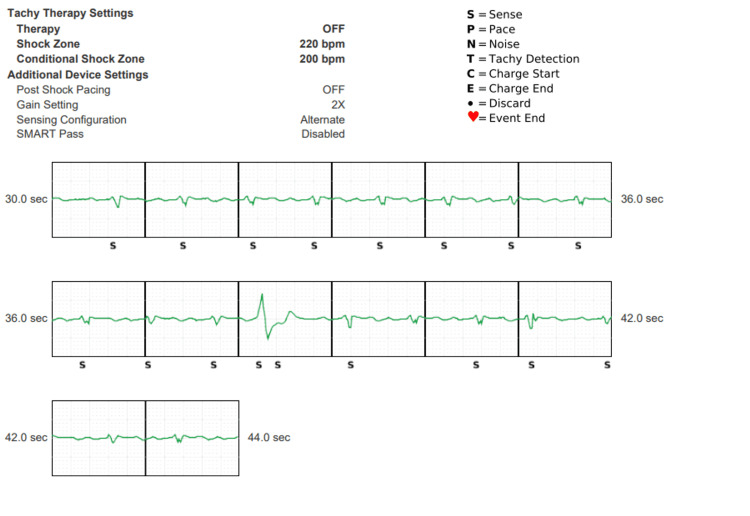
The vector being set as an alternate Here the S-ICD tracings demonstrated that even with this setting, there were still events of oversensing of S and T waves though there were no discharges. Undersensing is now noted. S-ICD - subcutaneous implantable cardioverter defibrillator

Unfortunately, reanalysis of all vectors, including the alternate vector (Figure 3), also showed double counting of the QRS but also undersensing. Of note, the alternate vector was the implant vector setting, but also Smart Pass was deactivated within several months due to undersensing. The device was then programmed to the primary vector in follow-up. There were no adequate vectors to detect the QRS complex reliably, as shown in Figure 3.

As the patient's ejection fraction improved over time, there were more oversensing and undersensing events despite the SMART Pass filter and change of vector. Considering that the patient's LVEF had recovered and he had never required appropriate ICD therapy nor had a history of ventricular arrhythmias, the ICD was deactivated with plans for device removal as an outpatient. A wearable cardioverter defibrillator was not indicated due to his LVEF recovery and lack of prior ventricular arrhythmias. The patient was continued on guideline-directed medical therapy (GDMT) with carvedilol and sacubitril/valsartan. The patient's S-ICD was ultimately removed, and a loop recorder was implanted due to a history of prior atrial flutter status post-ablation for AF and VT monitoring. Post-operative recovery was unremarkable. The patient is doing well with no arrhythmias noted on his loop recorder.

## Discussion

The use of S-ICDs over traditional ICDs is becoming more common since their approval by the Food and Drug Administration (FDA) in 2012. S-ICDs are implanted in patients for prevention of SCD who meet the criteria for traditional ICD implantation but in whom there is no indication for cardiac resynchronization therapy, permanent bradycardia pacing, or in those with recurrent VT that responds to anti-tachycardia pacing [8]. Although there is no data to suggest the superiority of S-ICDs over the conventional ICD with regard to clinical outcomes, they are often selected in younger patients without pacing indications and in patients with venous access issues or infection risk [9].

One major limitation of S-ICDs is that while transvenous ICDs have over one hundred programmable parameters, S-ICDs have less than ten, including the sensing vector, detection of heart rate for conditional shock, therapy output, committed shock zones, and transient post-shock pacing [3].

**Figure 4:**
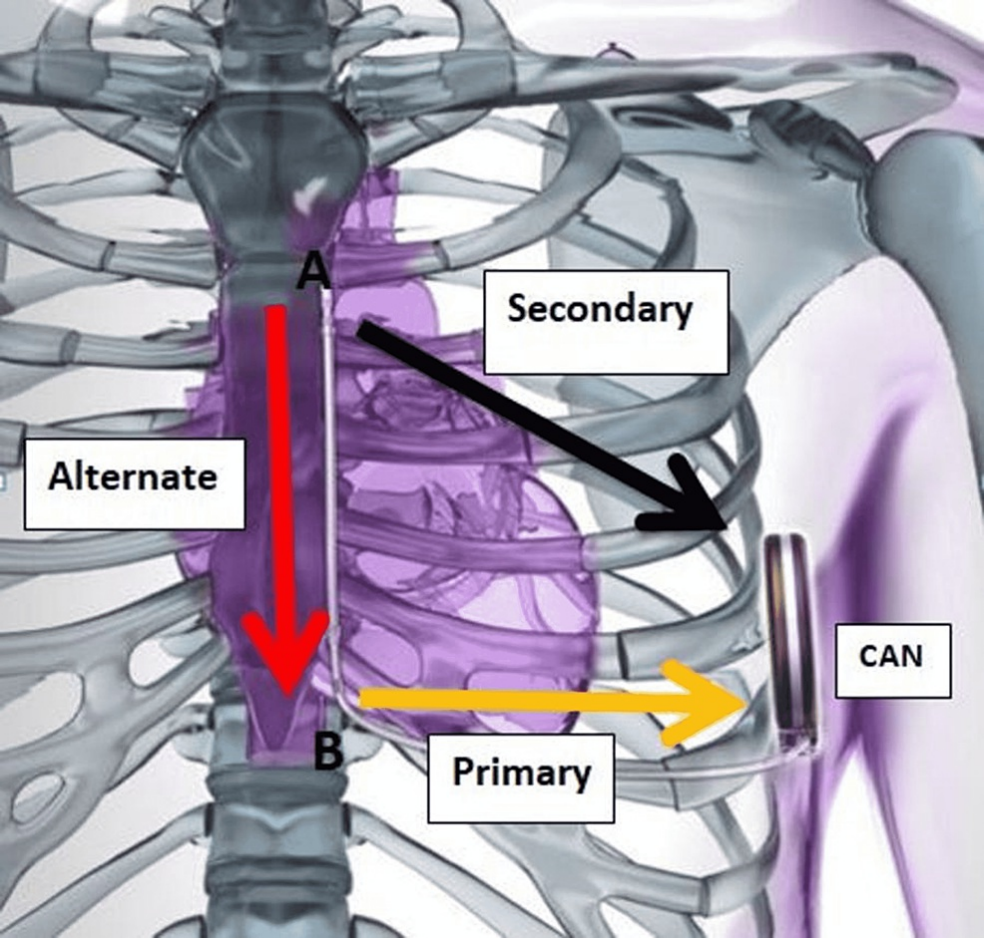
Schematic presentation of the subcutaneous ICD system position with primary, secondary, and alternate shocking vectors of the subcutaneous ICD system Image courtesy of Boston Scientific Corporation (Natick, USA), with permission to reprint. ICD - implantable cardioverter defibrillator

Both types of ICDs are known to carry a risk of inappropriate discharges. The incidence is reported to be 12-29% for those with S-ICDs [6]. The most commonly reported etiology for inappropriate discharges has been cardiac signal oversensing (73%), including T wave oversensing. Other causes include supraventricular tachycardias (SVTs), oversensing of low amplitude signals, and noncardiac sensing [10]. Additionally, cases have been reported due to subcutaneous air [7], generator change, and myopotential oversensing [9], as major noncardiac etiologies of oversensing. The basic components of an S-ICD involve a generator, electrodes, leads, and a coil, as shown in Figure 4 [3].

The mechanism of T wave oversensing relates to the T wave amplitude being similar to or larger than the QRS, causing the device to count two beats for every cardiac cycle [11]. If this persists for several cycles, the displayed rate increases and triggers a tachycardia alarm. S-ICD detects it as tachyarrhythmia events and, depending upon the programming, either delivers overdrive pacing or defibrillator discharges. Short and long QT syndromes, Brugada syndrome, exercise, cardiac sarcoidosis, electrolyte abnormalities, and hyperglycemia are the major etiologies of T wave oversensing described in the literature [11]. With respect to management, reprogramming of the device after the first reported clinical event of inappropriate discharges is mostly successful in preventing further episodes of inappropriate discharges in the future [10].

Our patient was diagnosed in 2016 with nonischemic cardiomyopathy that was initially attributed to aortic stenosis, but the reduced LVEF persisted after TAVR, which led to the implantation of the S-ICD in 2018 for primary prevention. The device was functioning well, and follow-up echocardiograms and multigated acquisition scans (MUGA) showed a pattern of improving LVEF (from 25-30 % in 2016, to 30-35% in 2017, to 55-60% in 2020). After recovery of his LVEF, our patient started experiencing inappropriate shocks. Initial reprogramming of the device with a change in sensing vectors and electrolyte repletion did not prevent further inappropriate discharges in our patient, as described in the literature [10].

Based on the current researched evidence in the literature, this is the first reported case of inappropriate S-ICD discharges due to T wave oversensing caused by the dramatic improvement in LVEF post-GDMT and cardiac remodeling, resulting in shifting of the heart to a more medial and anterior position leading to a change in the sensing of the vectors due to possible cardiac myopotentials causing T wave oversensing. One of the cases reported in 2016 mentioned a patient experiencing inappropriate shocks while lying on his left side, causing positional attenuation of R waves that increased the amplitude of cardiac signals resulting in oversensing [2]. Another case described T wave oversensing in hypertrophic cardiomyopathy leading to inappropriate shocks [11]. However, this was not present in this patient. 

While early studies suggested that S-ICDs can be used safely and effectively, there has been an increasing concern that these may be prone to inappropriate ICD shocks, which are associated with impaired quality of life, as well as increased mortality [1]. One reason could be that S-ICDs offer limited programming options as compared to transvenous devices when inappropriate shocks occur, and device extraction has been reported in 5% of patients owing to refractory oversensing issues [11]. The Smart Pass filtering algorithm was used in this patient, and it has been shown to reduce ICD shocks [12], but unfortunately could not overcome the sensing limitations in this patient. In our patient, as LVEF recovered to normal and the patient had no history of any ventricular arrhythmias, risks and benefits were discussed with the patient. It was decided to remove the S-ICD and implant a loop recorder. This unique clinical entity represents a learning opportunity for general cardiologists and electrophysiologists, increasing their awareness of this unusual etiology of T wave oversensing when ruling out possible other causes.

## Conclusions

Inappropriate ICD discharges can have a negative impact on patient outcomes. Clinicians should be aware that different etiologies of oversensing of S-ICD exist, which may lead to inappropriate discharges causing shocks. Careful analysis and interpretation of the different mechanisms responsible for inappropriate discharges may help to determine the most appropriate sensing vector. Inappropriate shocks should be managed carefully by eliminating the mechanism responsible for shock, which includes reprogramming of the device, higher shock thresholds, and adjustment of the sensing vector. If the appropriate management fails to control inappropriate shocks, device explantation may carefully be done in selected patients with implantation of another type of ICD or loop recorder depending upon the indication.
